# Supplementary material for: Integrative Transcriptome and Metabolome Analysis Reveals Candidate Genes Related to Terpenoid Synthesis in Amylostereum areolatum (Russulales: Amylostereaceae)
Source: J Fungi (Basel). 2025 May 16;11(5):383. doi: 10.3390/jof11050383 (PMC12113409; doi:10.3390/jof11050383)
Supplement: Supplementary file 1 [file jof-11-00383-s001.zip › jof-3589885-supplementary/Figure S1. LC-MS Total ion chromatograms (TIC) acquired from Amylostereum areolatum.pdf]

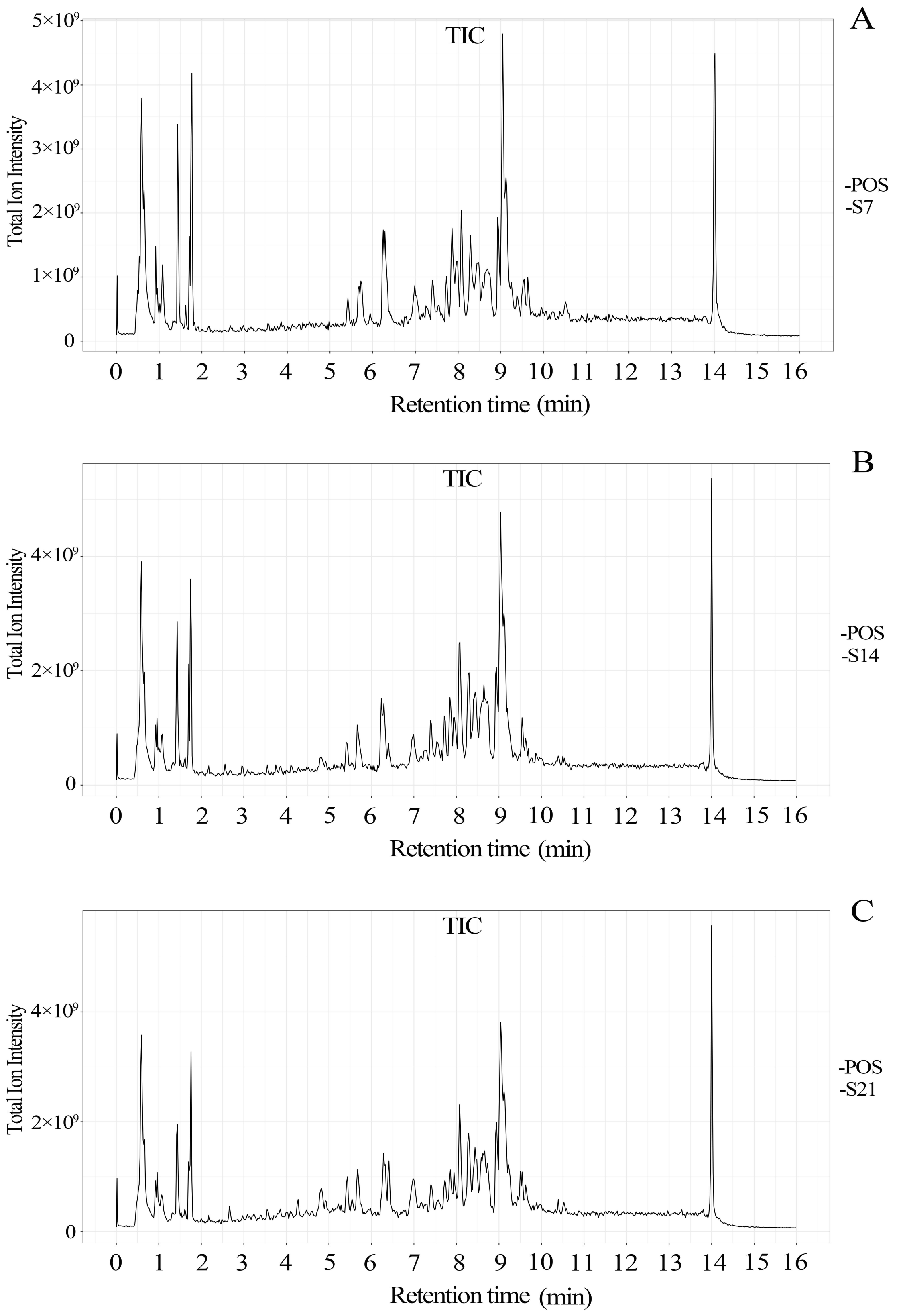
A
TIC
5×10⁹
4×10⁹
3×10⁹
2×10⁹
1×10⁹
0
Total Ion Intensity
0 1 2 3 4 5 6 7 8 9 10 11 12 13 14 15 16
Retention time (min)
-POS
-S7
B
TIC
4×10⁹
2×10⁹
0
Total Ion Intensity
0 1 2 3 4 5 6 7 8 9 10 11 12 13 14 15 16
Retention time (min)
-POS
-S14
C
TIC
4×10⁹
2×10⁹
0
Total Ion Intensity
0 1 2 3 4 5 6 7 8 9 10 11 12 13 14 15 16
Retention time (min)
-POS
-S21

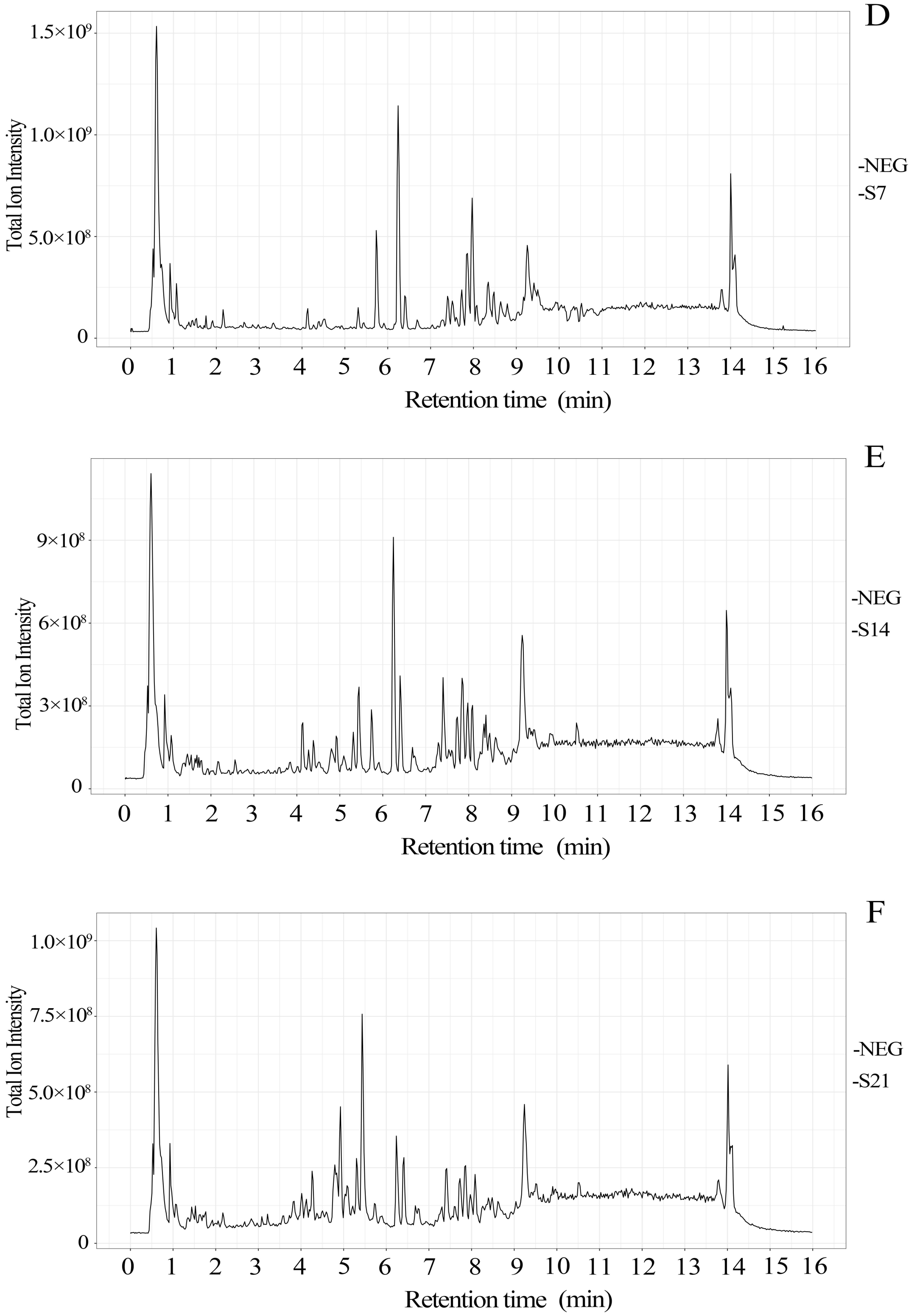

Figure S1. LC-MS Total ion chromatograms (TIC) acquired from *Amylostereum areolatum.* A-C represents the metabolic profiles of *A. areolatum* in the positive ion mode in samples at 7 days, 14 days, and 21 days, respectively. D-F represents the metabolic profiles of *A. areolatum* in the negative ion mode in samples at 7 days, 14 days, and 21 days, respectively.
