# Supplementary material for: Integrative Transcriptome and Metabolome Analysis Reveals Candidate Genes Related to Terpenoid Synthesis in Amylostereum areolatum (Russulales: Amylostereaceae)
Source: J Fungi (Basel). 2025 May 16;11(5):383. doi: 10.3390/jof11050383 (PMC12113409; doi:10.3390/jof11050383)
Supplement: Supplementary file 1 [file jof-11-00383-s001.zip › jof-3589885-supplementary/Figure S2. Volatile metabolic profiles of A. areolatum.pdf]

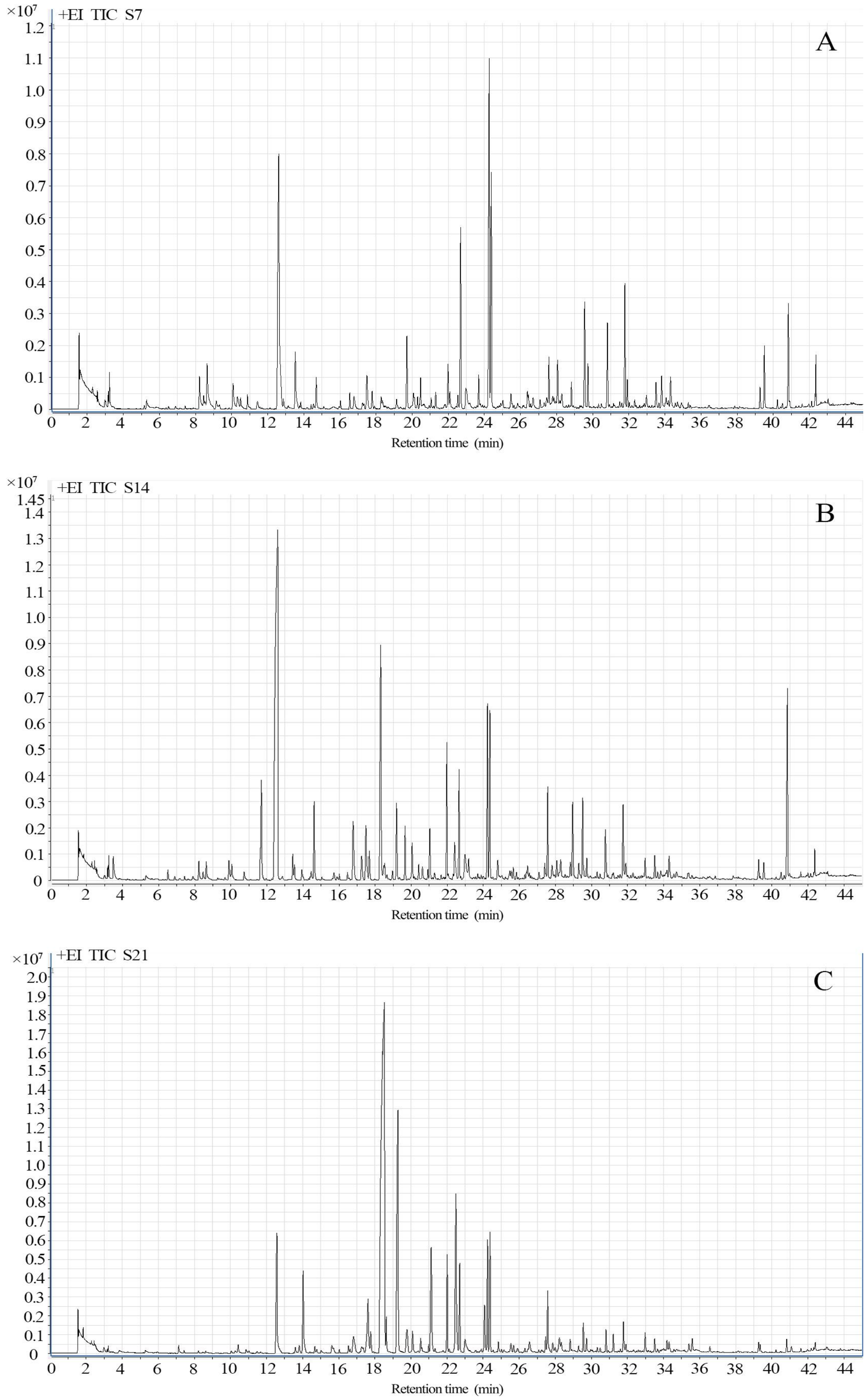

Figure S2. Volatile metabolic profiles of *A. areolatum.* (A)Volatile metabolic profiles of *A. areolatum* at 7-day, (B)Volatile metabolic profiles of *A. areolatum* at 14-day, (C)Volatile metabolic profiles of *A. areolatum* at 21-day.
