# Supplementary material for: Integrative Transcriptome and Metabolome Analysis Reveals Candidate Genes Related to Terpenoid Synthesis in Amylostereum areolatum (Russulales: Amylostereaceae)
Source: J Fungi (Basel). 2025 May 16;11(5):383. doi: 10.3390/jof11050383 (PMC12113409; doi:10.3390/jof11050383)
Supplement: Supplementary file 1 [file jof-11-00383-s001.zip › jof-3589885-supplementary/Figure S3. Clustering heatmap of terpenoid metabolites detected by LC-MS.pdf]

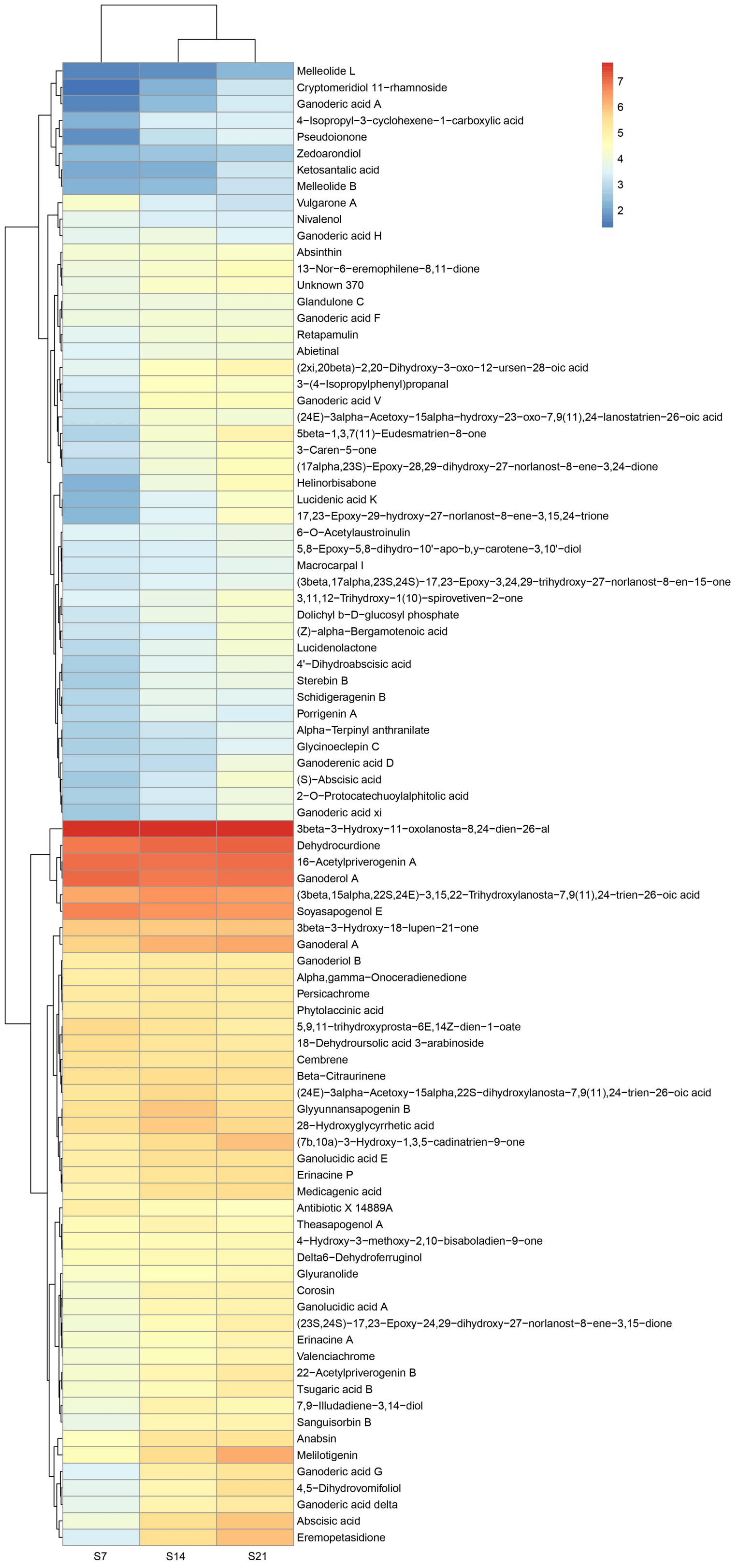

Figure S3. Clustering heatmap of terpenoid metabolites detected by LC-MS. S7, S14, S21: Sample of *A. areolatum* grown in damaged Mongolian Scots pine wood powder medium for 7day,14day, and 21day, respectively.
