# Supplementary material for: Integrative Transcriptome and Metabolome Analysis Reveals Candidate Genes Related to Terpenoid Synthesis in Amylostereum areolatum (Russulales: Amylostereaceae)
Source: J Fungi (Basel). 2025 May 16;11(5):383. doi: 10.3390/jof11050383 (PMC12113409; doi:10.3390/jof11050383)
Supplement: Supplementary file 1 [file jof-11-00383-s001.zip › jof-3589885-supplementary/Figure S4. Inter-sample orthogonal partial least squares-discriminant analysis detected by LC-MS.pdf]

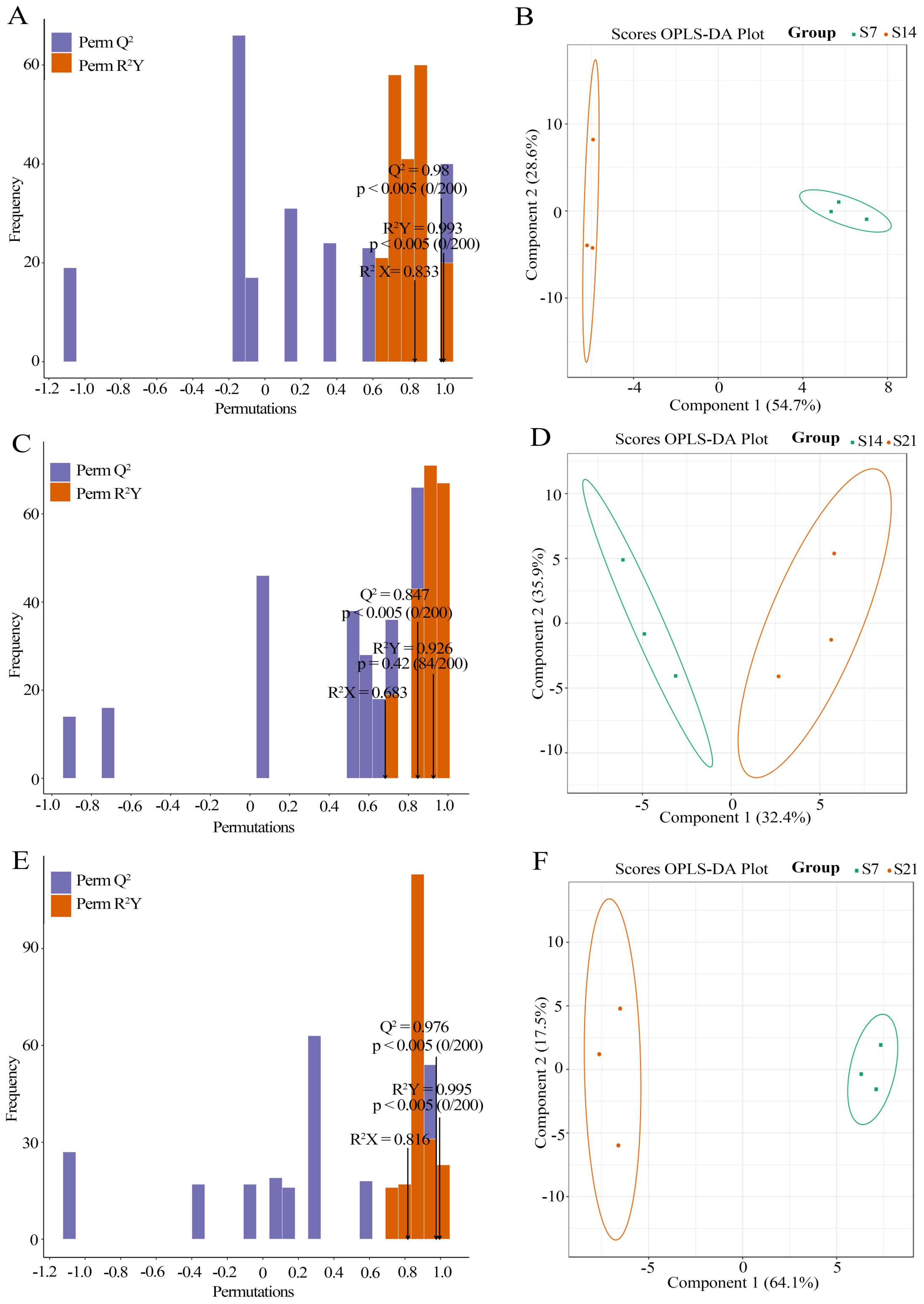

Figure S4. Inter-sample orthogonal partial least squares-discriminant analysis detected by LC-MS. A,B. OPLS-DA model plot and score plot for S14 vs. S7; C,D. OPLS-DA model plot and score plot for S21 vs. S14; E,F. OPLS-DA model plot and score plot for S21 vs. S7.
